# Factors associated with road traffic injuries in Tanzania

**DOI:** 10.11604/pamj.2016.23.46.7487

**Published:** 2016-02-19

**Authors:** Respicious Boniface, Lawrence Museru, Othman Kiloloma, Victoria Munthali

**Affiliations:** 1 Muhimbili Orthopaedic Institute (MOI), Dar es Salaam, Tanzania; 2 Injury Control Centre Tanzania (ICCT); 3 Muhimbili University of Health and Allied Sciences (MUHAS), Dar es Salaam, Tanzania; 4 University of Dar es Salaam, School of Health Sciences, Dar es Salaam, Tanzania

**Keywords:** Road traffic crashes, injuries, Tanzania

## Abstract

**Introduction:**

Injuries represent a significant cause of morbidity and mortality worldwide and road traffic crashes accounts for a significant proportion of these injuries. Tanzania is among the countries with high rates of road traffic crashes. The aim of this study was to determine the pattern, associated factors and management of road traffic injury patients in Tanzania.

**Methods:**

A cross-sectional study of patients involved in motor traffic crashes and attended in six public hospitals of Tanzania mainland between April 2014 and September 2014.

**Results:**

A total of 4675 road traffic injury patients were seen in studied hospitals, 76.6% were males. Majority (70.2%) were between 18 - 45 years age group. Motorcycles were the leading cause of road traffic crashes (53.4%), and drivers (38.3%) accounted for majority of victims. Fractures accounted for 34.1%, and injuries were severe in 2.2% as determined by the Kampala trauma score II (KTS II). Majorities 57.4% were admitted and 2.2% died at the casualty. Factors associated with mortality were; using police vehicles to hospital (P = 0.000), receiving medical attention within 2 to 10 hours after injury (P = 0.000), 18 - 45 years age group (P = 0.019), not using helmet (P = 0.007), severe injuries (P = 0.000) and sustaining multiple injury (P = 0.000).

**Conclusion:**

Road traffic Injuries in Tanzania are an important public health problem, predominantly in adult males, mostly due to motorcycle crashes. It is therefore important to reinforce preventive measures and pre-hospital emergency service is urgently needed.

## Introduction

Injuries represent a significant cause of morbidity and mortality in both developed and developing countries [1]. The Global Burden of Disease Study estimates that 10% of global deaths are due to injuries, and that if current trends persist, this burden will greatly increase in the next 20 years [2]. It is generally acknowledged that this problem is growing rapidly in Sub-Saharan Africa [3], and it is projected that by the year 2020, injuries in Africa will rank third among causes of disability adjusted life years [4]. Road traffic crashes account for much of the injury burden worldwide, especially in low and middle - income countries and are currently ranked 9th globally among the leading causes of disease burden, in terms of disability adjusted life years (DALYs) lost [5, 6]. By 2030, road traffic crashes are anticipated to rank in the top 3 of all cause disability behind depressive disorders and ischemic heart disease [5]. Accelerated urbanization and industrialization in many countries have led to an alarming increase in the rate of road traffic crashes. The number of vehicles has increased exponentially and has outpaced the development and adequate road space [7]. Developed countries have been able to control the number of fatal crashes through variety of countermeasures including occupant protection, better roads, effective trauma systems, and enforcement of traffic laws. However, less advancement has been made in reducing the number of injuries and fatalities due to traffic crashes within many lower and middle income countries. The total annual costs of road crashes to low-income and middle-income countries are estimated to be about US$ 65 billion, which is more than the total annual amount received in development assistance [8]. Road crashes in developing countries are more than double that of developed countries at 13.4/100,000 and 32.2/100,000 people in Europe and Africa respectively [5]. Tanzania being one of the African countries is heavily affected by road traffic crashes [9]. This is supported by national police data, which show that the number of road traffic injuries increased from 10107 in 1990 to 14548 in 2000, an increase of nearly 48%, at the same time the number of associated injuries increased by more than 44% while related fatalities by more than 64%. The risk of being killed in a traffic crash in Tanzania proportionate to the number of vehicles on the road is 20-30 times higher than in the USA and many countries in Western Europe [10], hence the WHO's Global Road Traffic Safety Report recommended a major focus on research and interventions in developing nations given over 90% of the world's fatalities on the roads occur in low-income and middle-income countries [11]. A hospital based injury surveillance involving six public hospitals in Tanzania, between November 2011 and December 2012 [12], revealed road traffic crashes to be the leading cause of injuries accounting for 47.5% of all injuries seen and 60.5% of injuries mortality, nevertheless pre-hospital care is almost nonexistent and health care service deliveries are poor. Furthermore, the request of public notification from police station before one is sent to a hospital has a tendency to delay patient further. Thus, many patients with severe injuries must be dying without medical care in Tanzania. This study was therefore aimed at investigating factors associated with road traffic injuries in Tanzania. The result of this study will help in developing control measures against this problem.

## Methods

*Study Setting*: data were collected from Muhimbili Orthopedic Institute, which is a national trauma referral hospital, and three city hospitals of Ilala, Mwananyamala and Temeke. These hospitals were chosen because they are the main Government hospitals serving the people of Dar es Salaam, capital city of Tanzania with a population of 4,364,541 [13]. Most of the city populations use these hospitals for medical care. Data were also collected from Tumbi hospital which is the main public hospital of Coast region. Coast region has a population of 1,098,668, and Tumbi hospital is located in Kibaha district which is traversed by one of the busiest highway in the country. The other study area included Morogoro regional hospital. Morogoro region has a population of 2,218,492 and is also located along the busiest highway in the country. These are public hospitals and a majority of residents seeking medical care visit these facilities. Data were also collected from the Police Traffic headquarters of Dar es Salaam, Coast and Morogoro regions. This was done in order to capture the number of road traffic crashes and victims who died at the site or before reporting to hospitals studied during the study period.

*Study design*: a prospective cross-sectional study was conducted

*Study population:* the study population included road traffic crash victims (attending the hospitals, dead on their way to the hospital or at the site of the crash) between 1^st^ April 2014 and 30^th^ September 2014. Data collection and management: A structured questionnaire was used by trained research assistants to collect information from the study participants in a face-to-face interview, and from health facilities medical records. Information was collected on socio-demographic characteristics, nature of injury, alcohol use, pre-hospital and hospital management offered and patient's outcome (Appendix 1). Severity of the injury was assessed using the Kampala Trauma Score 11 (KTS 11). Kampala Trauma Score is a scale for assessing severity of injury based on a combination of systolic blood pressure and respiratory rate on arrival, neurological status, seriousness of the injury and patient's age [14]. It is simplified so that it can be determined in outpatient settings of hospitals with limited resources. Information collected from regional traffic police headquarters was number of road traffic crashes, injuries, fatalities and presumed or known cause of the crash. Data were double-entered into Epi info version 3.5.1 (CDC, Atlanta, USA). Statistical analysis was conducted using STATA version 11 (Statacorp, College Station, USA).

*Statistical analysis*: all variables were categorized and described using frequency distribution. The dependent variable mortality was categorized as a dichotomous variable (death or no-death). Bivariate associations were described using chi-square tests. For those variables with observed frequency less than five fisher's exact test was applied. A variable with (p ≤ 0.05) with mortality was considered to be statistically significant.

*Ethical consideration*: ethical approval for the study was obtained from the Muhimbili University of Health and Allied Sciences Research Ethics Committee. An informed written consent was sought from patients or relatives.

## Results

### Police

Data obtained from police revealed 5869 road traffic crashes in six months April to September 2014, in three regions studied with 515 fatalities (died at the site of crash). Rates per 100,000 populations revealed Dar es Salaam to be leading in road traffic crashes and casualties, while fatalities were more in Coast region (Table 1). Reasons for road traffic crashes as stated by police were reckless/dangerous driving 40.6%, careless motorcyclists 26.4%, bad roads 17.9%, defective motor vehicles 6.9% and others (like careless cart pushers, animals crossing the roads) 8.2%

**Table 1:** Distribution of road traffic crashes, fatalities and injuries by regions surveyed (rates/100,000)

Region	April - September 2014		
	RTA	Death	Casualties
Dar es Salaam	115.7	6.4	79.1
Coast	27.6	12.7	49.1
Morogoro	23.2	4.3	28.2

Source; regional traffic police headquarters

### Hospitals

#### Demographic profile

A total of 4675 road traffic injury patients were seen between April 2014 and September 2014. The distribution of injury patients by hospital was Muhimbili Orthopedic Institute 30.5%, Morogoro 22.6%, Tumbi 13.8%, Temeke 12.7%, Amana 10.6% and Mwananyamala 9.9%. Male were 3581/4675 (76.6%) and female 1094/4675 (23.4%), with a male to female ratio of 3:1. Majorities (70.2%) belonged to the productive age group (18 to 45 years). Unemployed people accounted for the biggest proportion (28%), followed by business owners 27.3%, employed people 25.8% and students/pupils 18.2%. Most of the injured had primary school education 60.9%, followed by secondary education 27.6%.

#### Circumstances of the injury

Motorcycle (53.4%) was responsible for the majority of road traffic crashes, followed by motor-vehicles (42.5%) and bicycles (3.7%). Drivers (38.3%) accounted for the majority of victims, followed by passengers (35.4%), Pedestrians (25.5%) and unknown were (1.1%). Majority of motor-cycle crash victims were drivers (62.8%), passengers and pedestrians were more involved in motor-vehicles crash with 53.4% and 48.7% respectively. Helmet and seat belt use among motorcyclists and occupants of vehicles were recorded in 43.3% and 24.4% of patients respectively. History of alcohol consumption prior to the accident was reported in (21.7%) patients. Mode of transport to hospitals were (38.5%) by private vehicles, ambulances (36.3%), police (16.9%), bicycle/motorcycle (4.5%) and (4.1%) patients walked. Of the 4675 patients seen, 57.4% were admitted, 40.4% treated and sent home and 2.2% died at the casualty.

#### Nature of injury

Fractures were the most common nature of injury sustained accounting for 34.1%, followed by superficial injuries 26.1%. Multiple injuries occurred in (21.7%) patients and 15.4% sustained head injury (Table 2). Motor-cycle crashes accounted for majority of victims with head injury (54.3%), fractures (52.9%) and multiple injuries (51.2%). 80.2% of motor-cycle crash victims with head injury had no helmet. According to Kampala Trauma Score II (KTS II), the majority of patients sustained mild injuries 3908/4675 (83%). Severe injuries and moderate injuries were 86/4675 (2%) and 682/4675 (15%) respectively.

**Table 2:** Nature of injury by hospital

Nature	Amana N(%)	MOI N(%)	Morogoro N(%)	Mwananyamala N(%)	Temeke N(%)	Tumbi N(%)	Total N (%)
Abdominal	0 (0)	2 (0.1)	34 (3.2)	5 (1.1)	1 (0.2)	0 (0)	42(0.9)
Chest	2 (0.4)	5 (0.4)	69 (6.6)	5 (1.1)	5 (0.8)	5 (0.8)	91(1.9)
Fractures	97 (19.9)	855(60.2)	289(27.4)	155 (33.5)	63(10.6)	129(20.1)	1588(34.1)
Head Injury	31 (6.4)	412(28.9)	142(13.5)	60 (12.9)	36 (6.1)	35 (5.4)	716(15.4)
Multiple injury	162(33.3)	117 (8.2)	383(36.4)	111 (23.9)	81(13.7)	155(24.1)	1009(21.9)

NB: Fracture = extremities fracture; Multiple injury = if injury involved more than one part of the body

### Management

#### Pre-hospital care

Only 73/4665 (2%) of the victims received some form of management at the crash site from good Samaritans. Management offered was splinting of fractures using pieces of wood, compression dressing to arrest bleeding using victim's clothes. 85/1694 (5%) of the victims who used ambulances to hospital did receive some form of management. Management offered in ambulance was intravenous line established, fluid administered and compression dressing to arrest bleeding using bandages. Those who used other means of transport to hospital didn't receive any management en route.

#### Hospital management

Hospital management of the victims was more non-operative 3439/4300 (79.9%), and 861/4300 (20.1%) had operative management. 468/861 (54.4%) of those who had operative management were attended at Muhimbili Orthopedic Institute. The majority of patients 2735/4972 (55%) received medical attention at hospital in within 2 to 10 hours after injury.

#### Factors associated with mortality

Factors associated with mortality (Table 3) were; those transported by police vehicles to hospital (P = 0.000), receiving medical attention within 2 to 10 hours after injury (P = 0.016), driver (P = 0.000), 18 to 45 years age group (P = 0.019), not using helmet (P = 0.007), sustaining multiple injuries (P = 0.000) and severe injuries (P = 0.000).

**Table 3:** Factors associated with mortality

Factor	Death N (%)	No death N (%)	P value
**Transport to hospital**			0.000**
Ambulance	24 (23.3)	1662 (36.7)	
Bicycle/Motorcycle	2 (1.9)	189 (4.1)	
Foot	0 (0.0)	192 (4.2)	
Police	57 (55.3)	724 (16.9)	
Private vehicle	20 (19.4)	1758 (38.9)	
Total	103 (100)	4525 (100)	
**Time to treatment**			0.016**
0 – 1 hour	42 (40.4)	1812 (39.9)	
2 – 10 hours	57 (54.8)	2492 (54.9)	
> 10 hours	5 (4.8)	227 (5)	
Total	104 (100)	4531 (100)	
**Road user category**			0.000**
Driver	41 (40.6)	1713 (38.3)	
Passenger	21 (20.8)	1587 (35.5)	
Pedestrian	30 (29.7)	1136 (25.4)	
Unknown	9 (8.9)	39 (0.9)	
Total	101 (100)	4475 (100)	
**Age (Years)**			0.019**
< 18	9 (8.8)	731 (16.2)	
18 – 45	71 (69.6)	3163 (70.1)	
> 45	22 (21.6)	617 (13.7)	
Total	102 (100)	4511 (100)	
**Nature of injury**			0.000**
Abdominal	0 (0.0)	41 (0.9)	
Chest	3 (2.9)	88 (1.9)	
Multiple injuries	48 (46.6)	948 (20.9)	
Fractures	8 (7.8)	1574 (34.8)	
Head injury	44 (40.8)	672 (14.9)	
Superficial injuries	0 (0.0)	1199 (26.5)	
Total	103 (100)	4522 (100)	
**Injury Severity**			0.000**
Severe	52 (50.0)	34 (0.8)	
Moderate	29 (27.9)	649 (14.3)	
Mild	23 (22.1)	3850 (84.9)	
Total	104 (100)	4533 (100)	
**Helmet use**			0.007**
Yes	9 (22.5)	801(43.9)	
No	31 (77.5)	1024(56.1)	
Total	40 (100)	1825(100)	

** P value ≤0.05; N = number

## Discussion

The vast majority of injured patients in this study 3581/4675 (76.6%) were males, with a male to female ratio of 3:1. This is similar to other studies in Africa and elsewhere, were young men are reported to be largest consumers of the hospital emergency trauma services [10, 15]. Male predominance in this study is due to their increased participation in high risk activities, in the city of Dar es Salaam for example, it is not unusual to see young men boarding and disembarking from moving city public buses. Similar to what has been observed elsewhere [15–20]; this study shows the majority of injured patients to be in the economically active group of 18 - 45 years who were also more likely to die than patients in other age groups. This is depleting the economically productive population, which bears a direct impact on individuals, families, communities and the country at large, hence a need for urgent public policy response with special reference to education, engineering and emergency care of road traffic crash victims [21]. In this study, motorcycles were responsible for the majority of road traffic crashes accounting for 2366/4665 (53.4%) of cases. Motorcycle crashes was also the leading cause of head injuries, fractures, multiple injuries and mortality, similar to what has been reported in previous studies [22, 23]. Helmet use among motor-cycle crash victims in this study however was low (43.4%) and majority of victims with head injury (80%) had no helmet. This is similar to other studies which have reported low helmet use among motorcyclists in developing countries [24–26]. Motorcycle use is becoming popular in Tanzania as it has become a cheaper and easier means of transportation in most cities [22]. However their use is characterized by non-helmet use by riders and passengers, passenger overload, speeding, poor enforcement of safety and use of alcohol and drugs [11, 22]. This calls for enhanced development of appropriate regulations, legislation, and enforcement of traffic rules for the protection of vulnerable road users. Drinking and driving appeared to be among the contributing factors to road traffic crashes in this study, as 21.7% of the road traffic crash victims were suspected to be under the influence of alcohol. It is an open secret that drivers drink and drive with impunity, many victims including drivers, passengers and pedestrians are then admitted in gross intoxicated alcohol situation levels. However, there is no mechanism for measuring blood alcohol content in Tanzania and breath analyzers are under-used by Tanzanian Police and this could be a source of under reporting. In comparison, in Zambia, it was found that 30% of killed drivers, pedestrians and cyclists had unacceptable level of alcohol in the blood [27]. Only 1989/4972 (40%) of injury victims were seen within one hour of injury in this study. The majority of patients (55%) received medical attention in 2 to 10 hours after injury and 5% were not seen for more than 10 hours. The golden hour is a symbol for pre-hospital care quality. This delay minimizes the chance of survival for severely injured patients, and those who were attended in within 2 to 10 hours after injury were more likely to die. There is therefore a need for the establishment of emergency ambulance service system in the country to minimize these delays.

Injured patients in Tanzania face several challenges. Most importantly is lack of a pre-hospital emergency care system which means that injured patients are likely to be picked up by Samaritan motorists. The pre-hospital care of trauma patient has been reported to be the most important factor in determining the ultimate outcome after the injury [22]. In Tanzania police are usually first responders, however they are not trained how to handle trauma patients especially severely injured ones during transport. This potentially increases the risk for such patients. The majority of patients had minor injuries 83%, and this suggests that most patients could have been treated in the primary level health facilities such as health centers. There should be a triage system to match injury severity to level of care, in order to minimize overcrowding of injury patients in secondary and tertiary care services. Most of the severely injured patients sustained multiple injuries (55.3%), or head injury (24.7%), and these patients needs good trauma management system with proper protocols of resuscitation including damage control surgery. In this study only 20.1% of patients had operative management, and more than half (54.4%) of those who had operative management were attended at one center, Muhimbili Orthopedic Institute. Tanzania lacks an organized trauma system at the present moment, serious efforts are needed to put in place a trauma care system including establishment of trauma registries in all hospitals that will capture timely, accurate, reliable good quality data that will inform injury prevention and improve trauma care [28, 29]. In considering the findings of this study it is important to bear in mind the following limitations: firstly, this was a hospital based study involving 6 public hospitals only, it may not reflect what is happening in other hospitals, public health centers and the general community. Secondly, data collectors not collecting all data and so some are missing. Thirdly, information bias from participants and data collectors may have affected the quality of data, use of poor quality police data, and the time frame of the study was short.

## Conclusion

The results of this study provide valuable insight into the nature of road traffic injuries that are prevalent in Tanzania hospitals. Many severe injured patients are not surviving to be seen at the hospitals and many minor injured patients are being cared for in hospitals. This information is important for policy makers so that appropriate triage can be organized. There is also a need for pre-hospital emergency care to ensure that severely injured patients arrive at hospitals as soon as possible. The majority of road traffic crashes are preventable and enforcement of safety will help in reducing the occurrence of crashes. There is a need for public awareness campaigns concerning road safety rules to reduce the occurrence of road traffic crashes as well as improvement of roads.

### What is known about this topic

- Road traffic crashes account for much of the injury burden in Tanzania.
- Majority of the injured victims are in the economically active age group of 18 - 45 years.
- Motorcycles are responsible for the majority of road traffic crashes.

### What this study adds

- This study provides valuable information into the nature of road traffic injuries encountered in Tanzania hospitals.
- It also reveals that many severe injured victims are not surviving to be seen at the hospitals, due to lack of pre - hospital care to ensure severely injured patients arrive at hospitals as soon as possible.
